# Plant-Pathogenic *Fusarium* Species

**DOI:** 10.3390/jof9010013

**Published:** 2022-12-21

**Authors:** Łukasz Stępień

**Affiliations:** Plant-Pathogen Interaction Team, Institute of Plant Genetics, Polish Academy of Sciences, Strzeszyńska 34, 60-479 Poznań, Poland; lste@igr.poznan.pl; Tel.: +48-61-655-0286

**Keywords:** fungal genetics, *Fusarium*, host resistance, mycotoxins, plant–pathogen interaction

## Abstract

*Fusarium* species are ubiquitous fungi, both saprotrophic and pathogenic to plants, animals and humans. They are also potent mycotoxin producers which makes them one of the most devastating plant pathogens. Mycotoxin biosynthesis and regulation has recently become one of the mainstream research topics, since knowledge concerning individual metabolic pathways became available and modern ’omics’ techniques allowed us to expand this even further. Independently, high-throughput sequencing methodology helped researchers gain insight into the complex phylogenetic relationships among closely related genotypes comprising *Fusarium* populations, species and species complexes. Molecular tools have so far been very powerful in species identification and phylogeny, as the great diversity of the *Fusarium* genus has forced scientists to continuously revise previously described taxons.

In the present Special Issue entitled “Plant-Pathogenic *Fusarium* Species“ ten research articles have been published concerning pathogens from the *Fusarium oxysporum* species complex (FOSC) [1,2,3,4]. Ling et al. re-sequenced the genome of *F. oxysporum* f.sp. *conglutinans* isolate from diseased cabbage and analysed the genome-wide SNP polymorphisms to track the diversity of the populations in China [1]. Furthermore, sequence data obtained for a diverse set of *F. oxysporum* f.sp. *asparagi* isolates from southern and northern Europe failed to distinguish the populations from different climate regions. Moreover, isolates from varying geographical origins showed similar pathogenicity towards asparagus plants [2]. Kurian et al. used a specific approach to evaluate the involvement of Ca^2+^ signalling machinery components in the regulation of the germ-tube formation, required for initial *F. oxysporum* colony establishment. These preliminary results may pave the way towards using molecular engineering to control the infection process through signalling pathway component management [3]. Sophisticated imaging techniques (*egfp-*tagged quantitative imaging) coupled with quantitative pathogen detection (qPCR) are often deployed to detect the early stages of infection in plant tissues and track the sequential spread of the pathogen in invaded organs, a strategy employed for *F. oxysporum* R1 identification in saffron roots and corms [4]. 

*Fusarium verticillioides* is an significant pathogen of maize occurring worldwide and limiting grain yield and quality. The Vietnamese *F. verticillioides* population was screened to evaluate the efficacy of the resistance components of two maize genotypes varying in susceptibility. Using a well-established procedure of gene expression profiling of the essential defence-related genes, Tran et al. showed that a higher expression may contribute towards the performance of more resistant maize genotypes [5]. *Fusarium* fungi can be dispersed in various ways, mostly by air, soil, water and seeds. One of the less frequent transmission routes include insects acting as vectors for plant pathogens. Recent study have proven that beetles from the species *Xylosandrus morigerus* can be effective vectors for the *Fusarium solani* species complex (FSSC), even though the two isolates showed a considerable level of diversity and were reported to be pathogenic to different plant species [6]. 

Bioinformatics has given insights into the evolutionary and functional divergence of fungal proteins playing specific roles during the interspecific interactions between *Fusaria* and other fungal species or plant hosts and has become a fundamental tool in functional studies, as employed in identifying CFEM (common in fungal extracellular membrane) protein domains or KP4-like (killer toxin-like) proteins of *Fusarium graminearum* [7,8]. The CFEM domain-encoding genes are expressed during wheat infection, which suggests their possible role as *F. graminearum* effectors [7]. Expression profiling of genes encoding KP4-like proteins revealed differential transcription depending on the experimental conditions. While all *Fgkp4l* genes were up-regulated during direct interaction between *F. graminearum* and *Trichoderma gamsii* (pathogen’s antagonist), only one ortholog was expressed during the interaction in wheat spikes. Interestingly, these genes were more common in *Fusarium* species with a broad host range than in the specialized ones [8]. 

Environmental factors have a significant impact on fungal physiology and compounds produced by plants, either primary or secondary metabolites, are among the most researched agents influencing fungal growth and mycotoxin biosynthesis. Therefore, various kinds of plant extracts are currently being examined for their antifungal properties. Among others, plant extracts from susceptible and resistant pea cultivars have been compared in terms of their components and influence on *Fusarium* growth and metabolism. All metabolites tested, including coumarin, spermidine, p-coumaric acid, iso-orientin, and quercetin were reported to inhibit the synthesis of fumonisin B_1_ and beauvericin but did not impair isolate growth [9]. A similar inhibitory effect of mandarin extract metabolites on *Fusarium* mycotoxins was noticed by Badr et al., namely deoxynivalenol (DON) and zearalenone (ZEN), produced by the *Fusarium culmorum* strain. However, pure standards of the individual compounds exerted a higher impact than the complex extracts made from fruit by-products [10]. 

In conclusion, constant progress in *Fusarium* research can be seen and is expected to develop even more in the future in all areas of fungal biology, pathology and toxicology, especially with the aid of modern techniques to uncover the mechanisms of secondary metabolic regulation and interspecific molecular communication.
